# Age‐associated dysregulation of protein metabolism in the mammalian oocyte

**DOI:** 10.1111/acel.12676

**Published:** 2017-10-10

**Authors:** Francesca E. Duncan, Susmita Jasti, Ariel Paulson, John M. Kelsh, Barbara Fegley, Jennifer L. Gerton

**Affiliations:** ^1^ Department of Anatomy and Cell Biology University of Kansas Medical Center Kansas City KS 66160 USA; ^2^ Stowers Institute for Medical Research Kansas City MO 64110 USA; ^3^ Electron Microscopy Research Laboratory University of Kansas Medical Center Kansas City KS 66160 USA; ^4^ Department of Biochemistry and Molecular Biology University of Kansas Medical Center Kansas City KS 66160 USA; ^5^ Present address: Department of Obstetrics and Gynecology Feinberg School of Medicine Northwestern University Chicago IL 60611, USA

**Keywords:** folliculogenesis, nucleolus, oogenesis, proteostasis, reproductive aging, ribosome

## Abstract

Reproductive aging is characterized by a marked decline in oocyte quality that contributes to infertility, miscarriages, and birth defects. This decline is multifactorial, and the underlying mechanisms are under active investigation. Here, we performed RNA‐Seq on individual growing follicles from reproductively young and old mice to identify age‐dependent functions in oocytes. This unbiased approach revealed genes involved in cellular processes known to change with age, including mitochondrial function and meiotic chromosome segregation, but also uncovered previously unappreciated categories of genes related to proteostasis and organelles required for protein metabolism. We further validated our RNA‐Seq data by comparing nucleolar structure and function in oocytes from reproductively young and old mice, as this organelle is central for protein production. We examined key nucleolar markers, including upstream binding transcription factor (UBTF), an RNA polymerase I cofactor, and fibrillarin, an rRNA methyltransferase. In oocytes from mice of advanced reproductive age, UBTF was primarily expressed in giant fibrillar centers (GFCs), structures associated with high levels of rDNA transcription, and fibrillarin expression was increased ~2‐fold. At the ultrastructural level, oocyte nucleoli from reproductively old mice had correspondingly more prominent fibrillar centers and dense fibrillar centers relative to young controls and more ribosomes were found in the cytoplasm. Taken together, our findings are significant because the growing oocyte is one of the most translationally active cells in the body and must accumulate high‐quality maternally derived proteins to support subsequent embryo development. Thus, perturbations in protein metabolism are likely to have a profound impact on gamete health.

## Introduction

Aging is associated with cellular and tissue deterioration and is a prime risk factor for chronic diseases and declining health (Lopez‐Otin *et al*., 2013). The female reproductive system is unique in that it ages decades prior to other organ systems in the human body. Female reproductive aging is first characterized by a decline in egg quantity and quality that begins when women reach their mid‐thirties (advanced reproductive age) followed by a complete cessation of reproductive function at menopause (Broekmans *et al*., 2009). Reproductive aging results in loss of fertility and endocrine function and is becoming a significant societal problem as women globally are delaying childbearing (Heffner, 2004; Johnson *et al*., 2012). Women of advanced reproductive age have an increased risk of miscarriage, birth defects, and twinning (Heffner, 2004; Johnson *et al*., 2012). Moreover, the altered endocrine environment associated with reproductive aging has broad impact on general health because ovarian hormones, such as estrogen, drive cardiovascular, immune, cognitive, and bone functions (Traub & Santoro, 2010). In fact, menopause has been shown to accelerate aging (Levine *et al*., 2016). Such general health consequences of reproductive aging may become even more tangible as the gap between menopause and lifespan continues to widen. Thus, there is a need to fully understand the cellular and molecular mechanisms underlying reproductive aging.

Reproductive age‐associated infertility is primarily due to defects that occur at the level of the gamete, and this phenomenon is best exemplified by data from assisted reproductive technology cycles. For example, if women use their own gametes to conceive, there is a strong maternal age effect such that the likelihood of having a live offspring decreases with age (Check *et al*., 2011). In contrast, if women use donor eggs from young, healthy individuals to conceive, the maternal age effect is effectively negated (Check *et al*., 2011). Thus, the biological age of the egg significantly determines quality and impacts reproductive outcomes.

Gamete quality is dictated by cytoplasmic and meiotic competence acquired during oocyte development (Eppig, 1996). Cytoplasmic competence refers to the accumulation of key maternal factors during the oocyte's growth phase, which is highly active during the primary and secondary follicle stages. These maternal products include mRNAs, proteins, and organelles, which support meiotic maturation, fertilization, and early preimplantation embryo development (Gosden & Lee, 2010). This maternal legacy is essential, as developmental events prior to the activation of the zygotic genome occur largely in the absence of transcription, and the stored cellular components of the sperm do not contribute significantly to cleavage‐stage embryogenesis. Meiotic competence instead refers to the ability of a fully grown oocyte to resume the cell cycle in response to hormonal cues and undergo meiosis, or two sequential rounds of cell division without an intervening round of DNA replication, to produce a haploid gamete (Hunt & Hassold, 2008). Several studies have demonstrated significant age‐associated changes in processes related to both meiotic and cytoplasmic competence. For example, the incidence of egg aneuploidy increases with advanced reproductive age due to alterations in chromosome structure, chromosome‐associated proteins, cell cycle regulation, and the spindle machinery (Hunt & Hassold, 2008; Jones & Lane, 2013; Hornick *et al*., 2015; Nakagawa & FitzHarris, 2017). In addition, mitochondrial dysfunction is one of the most prominent cytoplasmic changes that occurs with age in the oocyte (Seidler & Moley, 2015). Together, these observations underscore the notion that the age‐associated decline in gamete quality is complex and multifactorial.

The goal of the current study was to perform single‐follicle RNA‐Seq on follicles isolated from reproductively young and old mice to shed further insight on the cellular processes and molecular determinants that are affected by age in the oocyte during its active growth phase – a critical window in female gamete development. This unbiased approach revealed molecular patterns of aging at an unprecedented level. Genes involved in many previously identified age‐dependent functions were found to be differentially expressed as expected, including those related to chromosomes, the spindle, microtubules, kinetochores, and mitochondria. However, we identified a new signature that indicates that proteostasis is disrupted with age, with gene expression differences related to the nucleolus, the endoplasmic reticulum, and protein processing. To validate this observation, we performed a detailed analysis of nucleolar architecture and function in oocytes from reproductively young and old mice, as this organelle is responsible for ribosome biogenesis and its structure is intimately related to its function (Olson & Dundr, 2005). We examined the protein expression profiles of essential nucleolar proteins along with nucleolar ultrastructure in oocytes and found that advanced reproductive age was associated with structural changes indicative of disrupted function. Oocytes from mice of advanced reproductive age also had increased ribosome numbers and expression of the ribosomal protein, RPS2. Taken together, our results suggest that age‐associated changes in nucleolar structure and protein metabolism could contribute to quality decline in oocytes.

## Results

### Coordinated oogenesis and folliculogenesis is disrupted with advanced reproductive age

**Figure 1:**
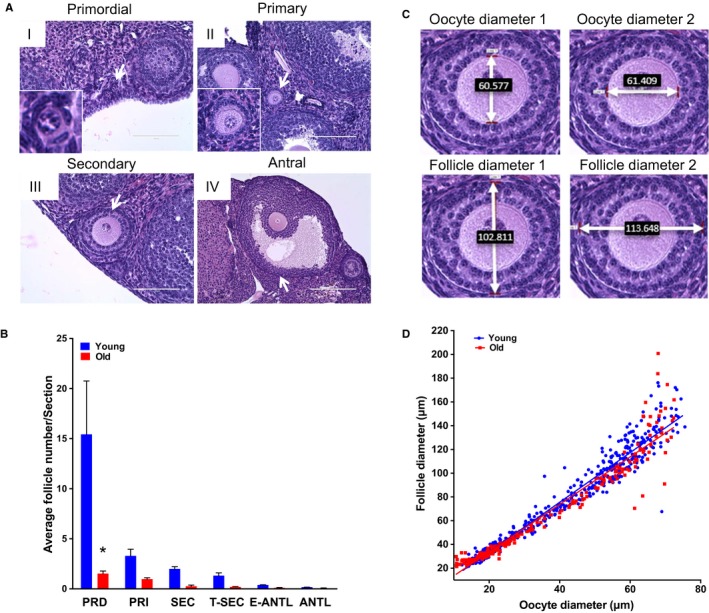
Reproductive aging in CB6F1 mice is associated with reduced ovarian reserve and altered follicle growth dynamics. (A) Representative hematoxylin and eosin (H&E)‐stained ovarian tissue sections demonstrate follicle‐stage classes according to histologic morphology (see Fig. S1 for additional images). White arrows highlight the particular follicle class. Insets show magnified images of primordial and primary follicles. Scale bars are (I–III) 100 μm and (IV) 200 μm. (B) Graph showing the average number of each follicle class per ovarian section (every fifth section of serially sectioned ovaries was counted; *N* = 3 ovaries from reproductively young and old mice). PRD: primordial; PRI: primary; SEC: secondary; T‐SEC: transitioning secondary; E‐ANTL: early antral; ANTL: antral. Two‐way anova was performed; asterisks denote *P* < 0.001. (C) Images showing how oocyte and follicle diameters were determined by the mean of two perpendicular measurements on H&E‐stained ovarian tissue sections at 40× magnification. (D) Mean oocyte and follicle diameters were plotted for each individual growing follicle (primary–transitioning secondary). Each dot shows the mean oocyte diameter on the *x*‐axis, and its corresponding follicle diameter on the *y*‐axis. For each ovary, a minimum of 50 follicles in each stage were measured. Pearson's correlation and linear regression were performed. *R* ^2^ for young and old were 0.9455 and 0.9256, respectively. The slopes between age cohorts were significantly different (*P* = 0.039).

Physiologic aging in several mouse strains phenocopies reproductive aging phenotypes in humans. The CB6F1 mouse strain, for example, exhibits increased egg aneuploidy and ovarian stromal fibrosis with advanced reproductive age (Chiang *et al*., 2010; Briley *et al*., 2016). To further validate the CB6F1 mouse strain as a suitable reproductive aging model, we harvested ovaries from reproductively young and old mice and performed follicle counts by routine histological analysis to evaluate the ovarian reserve (Bristol‐Gould *et al*., 2006). Follicles were classified according to developmental stage, and the average number of each follicle class per histological section for each ovary was determined (Fig. 1A,B and Fig. S1A). We observed a significant reproductive age‐associated reduction in the number of primordial follicles, with reproductively old mice having an average of 1.5 ± 0.46 primordial follicles per histological section compared to 15.4 ± 9.24 in young counterparts (Fig. 1B). Reproductively old mice also exhibited a reduction in all other follicle classes, which comprise the activated and growing pool, but this trend was not significant (Fig. 1B). Thus, the CB6F1 strain undergoes a prominent age‐associated decrease in the ovarian reserve as occurs in humans and contributes to a decline in fertility (Broekmans *et al*., 2009).

Oogenesis and folliculogenesis are tightly coordinated and rely on bidirectional communication between the oocyte and its surrounding somatic cells, and the oocyte orchestrates the rate of follicle development (Eppig *et al*., 2002). To determine whether reproductive aging is associated with differences in coordinated oocyte and follicle growth dynamics, we measured the mean diameters of individual follicles and their corresponding oocytes on histological ovarian sections (Fig. 1C and Fig. S1B,C). We analyzed primary to transitioning secondary‐stage follicles, as these developmental stages correspond to periods of active oocyte growth that occur independently of gonadotropin regulation. When follicle diameters were plotted against oocyte diameters for each individual follicle, we observed a strong positive linear correlation, as expected, such that as the follicle diameter increases, so does the oocyte diameter (Fig. 1D). However, these growth trajectories were significantly different between follicles isolated from reproductively young and old mice (Fig. 1D). Such differences were attributed to the observation that oocytes within secondary follicles of similar diameters (89.77 ± 2.533 μm and 84.16 ± 1.552 μm, *P* > 0.05) were significantly larger (50.99 ± 1.212 μm vs. 47.33 ± 0.9169 μm, respectively, *P* < 0.0001) in reproductively old mice compared to young mice (Fig. S1B,C). Although the observed differences may appear small, they are significant, and diameter differences translate into pronounced volumetric changes. As oocytes orchestrate the rate of follicle development, our data suggest that there are likely intrinsic age‐associated differences in cellular processes in the growing oocyte that warrant further investigation.

### Reproductive aging is associated with increased variability in follicle gene expression

**Figure 2:**
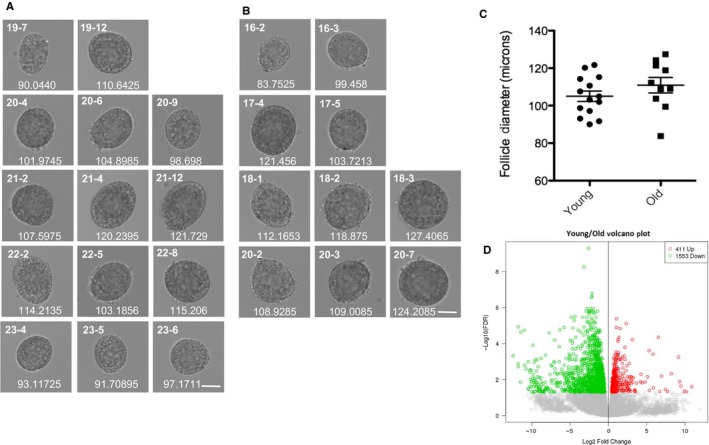
RNA‐Seq on individual follicles from reproductively young and old mice reveals differential gene expression despite indistinguishable morphology. Brightfield microscopy images of all follicles from reproductively (A) young and (B) old mice, which were processed for individual follicle RNA sequencing and analysis. Each row shows follicles that were isolated from an individual mouse. Each follicle's unique identifier used throughout the manuscript is listed at the top left corner of each image. The mean diameter (μm) for each follicle, determined by taking the mean of two perpendicular measurements, is shown at the bottom of each image. The scale bar is 50 μm. (C) The graph shows the mean follicle diameter measurement of each follicle shown in (A) and (B). A *t*‐test was performed and there was no difference in diameters between the age cohorts (*P* = 0.2338). (D) Genes that are overall differentially expressed between follicles from reproductively young and old mice are shown in a volcano plot. Genes that are expressed more highly in young are shown in red (411), and those that are more highly expressed in old are shown in green (1553).

To obtain an unbiased view of cellular processes that may be altered with advanced reproductive age, we performed RNA‐Seq on individual growing follicles isolated from reproductively young and old mice. We performed RNA‐Seq on intact follicles because stromal fibrosis – particularly in ovaries from mice of advanced reproductive age – precludes efficient isolation of growing oocytes (Briley *et al*., 2016). Data were obtained from a total of 24 follicles, with 14 from five reproductively young mice and 10 from four reproductively old mice (Fig. 2A,B). All follicles used for RNA‐Seq had similar morphology and diameter (105.0 ± 2.77 μm vs. 110.9 ± 4.15 μm, young and old, respectively; *P* = 0.23; Fig. 2 2A‐C). Despite these macroscopic similarities, however, gene expression analysis revealed 1964 differentially expressed genes between follicles from reproductively young and old mice (Fig. 2D, Supporting Information). 1553 genes had significantly higher expression in follicles from reproductively old mice, whereas 411 genes had significantly higher expression in young counterparts (Fig. 2D).

**Figure 3:**
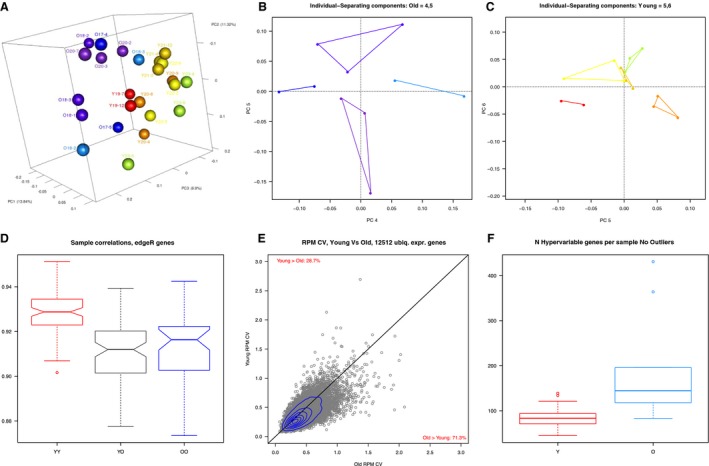
Principle component analysis can distinguish follicles from reproductively young and old mice and follicles from reproductively old mice exhibit greater variability in gene expression. (A) The first three components of PCA effectively separate the young samples (red, orange, yellow, green) from the old (blues and purples). Two follicles from reproductively old mice are closer to the young than the other old (O16.3, O17.5). (B) PCA components 4 and 5 separate follicles derived from each individual old mouse from each other. (C) PCA components 5 and 6 separate follicles derived from each individual young mouse from each other. (D) The pairwise comparisons of the Pearson's correlations, shown collectively in a box plot from young–young (YY), young–old (YO), and old–old (OO) samples, indicate that young samples are more highly correlated with each other than old samples. (E) For young and old samples, we compared CVs of normalized counts for genes detected in all samples. 71% of genes had higher values for old samples, indicating greater variability in gene expression with age. (F) We calculated the number of hypervariable genes per sample. The term ‘hypervariable’ indicates a gene with an extreme expression profile; that is, it has one rpkm *z*‐score > 4. For each sample, we asked how many hypervariable genes had highest expression. Young samples had < 100 on average, while old samples had close to 150 on average, indicating that age was associated with extreme expression.

Principle component analysis (PCA) was performed in R using log2 counts for protein‐coding genes, pseudogenes, and lncRNAs, with at least four counts in any sample to visualize the data by reducing the dimensionality while retaining the variation (Fig. 3A‐C). Follicles from reproductively young and old mice could be distinguished by the first three components, indicating that age accounts for the largest patterns of variation in gene expression in the dataset (Fig. 3A). We note that two of the reproductively old mice had individual follicles that were closer to the young cohort and farther from the old cohort, suggesting differences in follicle quality within an animal and a biological continuum of reproductive aging (Figs 2B and 3A; O16‐3 and O17‐5). Variations in gene expression were also intrinsic to the mice themselves, as components 4–6 were sufficient to spatially resolve individual animals in the PCA analysis (Fig. 3B,C). Overall, the individual reproductively old mice separated on higher components (components 4 and 5) compared to individual young mice (components 5 and 6) due to their greater variation in follicle gene expression (Fig. 3B,C).

To follow up on the increased variability observed in follicles from reproductively old mice relative to young mice, we performed a correlation analysis among samples (Fig. 3D). The correlation between the gene expression profiles for all of the follicles showed a range of 0.87–0.95. Although correlations between young and old samples were lower than those between young and young, as would be predicted, they were equivalent to correlations between old and old (Fig. 3D). This indicates more heterogeneity in gene expression in follicles from reproductively old mice compared to young (Fig. 3D). This can be further appreciated by plotting the coefficient of variation (CV) for each gene in the young samples vs. the old; the follicles from reproductively old mice have more genes with higher CVs (Fig. 3E). Specifically, of 12512 genes detectable in all samples, 8918 (71%) had higher CVs in the old cohorts, whereas 3594 (29%) had higher CVs in young. In addition to increased variability in gene expression, follicles from reproductively old mice exhibited more genes with ‘hypervariable’ expression relative to follicles from young mice (Fig. 3F, Fig. S2A, Supporting Information). We defined a hypervariable gene as a gene with a log2‐ RPKM *z*‐score of ≥ 4 in any sample. These are not merely genes detected in only one sample; of 3094 hypervariable genes, 90% were detected in two or more samples, including 12% detected in all samples. 61% of these genes had maximum expression in old follicles.

### Gene expression signatures related to inflammation and protein quality control pathways distinguish young and old growing follicles

**Figure 4:**
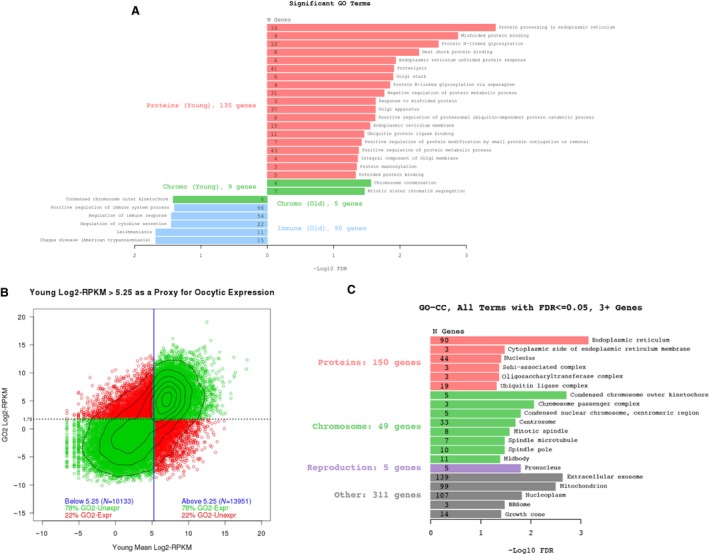
Genes involved in proteostasis are differentially expressed with age in the growing oocyte. (A) The butterfly plot shows the major trends in significantly enriched GO terms from the differentially expressed genes within the intact follicle. Terms enriched in young samples are on the right, and terms enriched in the old samples are on the left. The *x*‐axis is the –log10 of BH‐corrected *P*‐value. Numbers in bars indicate the total number of differentially expressed genes with the specified term. (B) Genes were assigned to ‘oocyte’ based on their expression in publicly available data. (C) Cellular component GO terms from the differentially expressed genes assigned to the oocyte reveal previously known age‐based signatures (i.e., mitochondria, spindle, microtubules, chromosomes) as well as a new category involved in proteostasis (red).

To further appreciate specific age‐associated follicle gene expression signatures, we examined the differentially expressed genes using KEGG pathway and GO term analyses (Table 1, Fig. 4A and Supporting Information). Despite follicles from reproductively old mice exhibiting a > 3.7‐fold increase in differentially expressed genes compared to the young cohort, only two KEGG pathways and five GO terms were significant in this population (Table 1, Fig. 4A, Supporting Information). Interestingly, these gene categories largely related to immune function and chemokine signaling, consistent with the known increased inflammation that occurs with advanced reproductive age (Briley *et al*., 2016). In follicles from reproductively young mice, four KEGG pathways were significantly upregulated, including N‐glycan biosynthesis, metabolic pathways, estrogen signaling, and protein processing in the endoplasmic reticulum (ER) (Table 1). We were especially intrigued by the term ‘protein processing in the ER’, as protein quality control is often affected in aging but has not been reported to be associated with female reproductive aging (Kaushik & Cuervo, 2015).

**Table 1:** KEGG Pathway results of differentially expressed genes in individual follicles from reproductively young and old mice

KEGG term name	Genes
Gene pathways enriched in follicles from young mice	
Metabolic pathways	Uck1; Impdh1; Cyp17a1; Ptgis; Atp5g3; Pfas; Umps; St6gal1; Cbs; Man2a1; Psat1; Cndp2; Minpp1; Papss2; Atp5b; Nnt; Gls; Ahcy; Rpn2; Hsd3b1; B4galt1; Ppcs; Mogs; Rpn1; Idh2; Me3; Prps1; Gpt2; St3gal4; Acsbg1; Cyp11a1; Gclc; Chpf2; Pold1; Pck2; Cyp2s1; Mgat2; B3galt6; Ptges3; Alg6
Protein processing in endoplasmic reticulum	Prkcsh; Hsp90b1; Ssr1; Derl1; Hspa5; Rpn2; Mogs; Rpn1; Mbtps1; Uggt1; Sec24d; Ckap4; Hspa1b; Hspa1a
N‐Glycan biosynthesis	St6gal1; Man2a1; Rpn2; B4galt1; Mogs; Rpn1; Mgat2; Alg6
Estrogen signaling pathway	Hsp90b1; Hbegf; Creb1; Kcnj3; Fkbp4; Creb3l3; Hspa1b; Hspa1a
Gene pathways enriched in follicles from old mice	
Chagas disease (American trypanosomiasis)	Ccl3; Tgfb3; Ppp2r2b; Gna14; Fas; Pik3r3; Cd3d; Ccl12; C1qc; C1qb; Tlr4; Tgfb2; Mapk11; Gnai1; Mapk3
Leishmaniasis	Tgfb3; H2‐Oa; Jak2; Ptgs2; H2‐Aa; Tlr4; Tgfb2; Mapk11; Fcgr4; Fcgr3; Mapk3

GO term analysis also corroborated the observation that protein quality control mechanisms are enhanced in follicles from reproductively young mice, as we found a striking enrichment of gene categories (biological processes, cellular components, and molecular function) that are involved in protein modification and metabolism in young follicles (Fig. 4A and Supporting Information). Specifically, these categories included protein mannosylation, N‐linked glycosylation, ubiquitination, heat shock protein binding, the unfolded protein response, and misfolded protein response. In addition, gene expression differences occurred in genes relevant to the ER and Golgi (Fig. 4A and Supporting Information). Together, this analysis suggests that young follicles may have more stringent protein quality control mechanisms than old follicles.

### Age‐associated oocyte‐specific gene signatures highlight alterations in protein metabolism

Because our RNA‐Seq analysis was performed on intact follicles, we could not readily distinguish the relative oocyte vs. granulosa cell contribution to the observed gene expression patterns. Therefore, we performed a post hoc *in silico* analysis of our dataset to parse out these relative contributions. To do this, we used a publically available gene expression database obtained from pools of oocytes at a similar developmental stage as our study, which allowed us to generate an RPKM cutoff value that best indicated a gene's likelihood of being derived specifically from the oocyte (Fig. 4B; Veselovska *et al*., 2015). We then filtered our differentially expressed genes according to this threshold and found that overall 48% of genes had likely origins from the granulosa cells compared to 52% genes from the oocyte. We were particularly interested in the nature of the oocyte‐specific changes that occurred with advanced reproductive age. Interestingly, in follicles from reproductively old mice, we observed a marked upregulation of three critical oocyte‐specific genes (Fgf8, Gdf9, and Bmp15) that encode paracrine factors that stimulate metabolic cooperativity with the granulosa cells (Supporting Information; Knight & Glister, 2006; Sugiura *et al*., 2007). Thus, these results suggest that the aging oocyte is sending out increased signals, perhaps reflecting decreased support from the somatic microenvironment.

We repeated the GO term analysis on this filtered gene list to further pinpoint the gene expression signatures that were likely to be derived from the oocyte. We found that differentially expressed genes were enriched for terms associated with many of the key cellular components known to deteriorate with age in oocytes, including microtubules, kinetochores, chromosomes, and chromosome‐associated proteins (Fig. 4C; Jones & Lane, 2013; Nakagawa & FitzHarris, 2017). These cellular components were also mirrored in the GO biological processes, which indicated enrichment of processes related to microtubules, cell division, and cell cycle (Fig. S2B). Thus, these findings affirm that these processes undergo age‐related changes in the oocyte and lend validity to our dataset. Due to the unbiased nature of our approach, we were also able to identify novel pathways that were differentially expressed with age. Interestingly, our data suggest that protein metabolism is an additional major pathway that is disrupted with age given that genes related to protein quality control (i.e., protein modification and the unfolded protein response) and related to cellular components involved in protein metabolism (i.e., nucleolus, ubiquitin ligase complex, and ER) were differentially expressed (Fig. 4C and Fig. S2B).

### Reproductive aging is associated with nucleolar architecture alterations in the growing oocyte

Given the strong, novel indication from the RNA‐Seq data that general protein metabolism pathways differ with age specifically in the oocyte, we performed a detailed comparative analysis of the nucleolus in growing oocytes from young and old mice (Fig. S3). We focused on the nucleolus, as it was identified as a cellular component that is altered with age, and it is the organelle responsible for ribosome biogenesis and thereby protein production (Fig. 4C). The architecture of the nucleolus is subcompartmentalized in three layers organized in a core–shell organization, with specific proteins in each layer. Ribosome assembly within the nucleolus is a vectorial process beginning most internal and moving outward as ribosomes are assembled (Olson & Dundr, 2005). In this manner, the steady‐state movement of nucleolar components during ribosome assembly determines the normal structure of an active nucleolus, and alterations in nucleolar architecture typically reflect defects in ribosome biogenesis (Olson & Dundr, 2005). We specifically examined the expression and distribution of three nucleolar markers that localize to distinct layers of the nucleolus and are indicators of the vectorial process as they function in early, mid‐, and late stages of ribosome biogenesis. These markers include (i) upstream binding transcription factor (UBTF), a Pol I cofactor necessary for rDNA transcription; (ii) fibrillarin, an rRNA methyltransferase; and (iii) nucleolin, a multifunctional protein that participates in late stages of ribosome biogenesis (Casafont *et al*., 2007; Abdelmohsen & Gorospe, 2012; Rodriguez‐Corona *et al*., 2015).

We first characterized the expression profiles of these markers in growing oocytes from young mice, and found that each nucleolar component had unique distributions (Figs S4 and S5). UBTF exhibited two expression patterns in the growing oocyte nucleolus (Fig. S5A‐I,II). In some oocytes, UBTF localized to multiple foci or punctae throughout the nucleolus in what are likely sites of Pol I bound to rDNA loops based on similar patterns in other cell types (Fig. S5A‐I; Casafont *et al*., 2007). Alternatively, UBTF was concentrated in large domains (Fig. S5A‐II), which are analogous to giant fibrillar centers (GFCs) reported in sensory ganglia neurons (Casafont *et al*., 2007). GFCs are associated with high levels of rDNA transcriptional activity and are also potential storage sites for factors essential for ribosome biogenesis (Casafont *et al*., 2007). Fibrillarin was expressed uniformly throughout the oocyte nucleolus, but its expression levels varied among oocytes (Fig. S5A‐III,IV). Nucleolin exhibited two different configurations (Fig. S5A‐V,VI). Nucleolin was either distributed evenly throughout the entire nucleolus in a ‘homogeneous’ configuration, or it was enriched at the nucleolar periphery in a ‘rim’ pattern (Fig. S5A‐V,VI).

**Figure 5:**
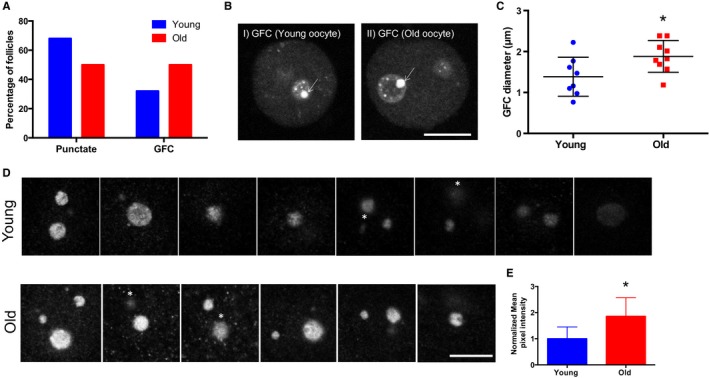
Reproductive aging is associated with altered nucleolar architecture. (A) The patterns of UBTF expression in oocyte nucleoli were examined in follicles isolated from reproductively young and old mice. The graph shows the distribution of UBTF conformations. Chi‐square analysis was performed and *P* = 0.0097. (B) Representative images of GFC size differences (arrows) in oocyte nucleoli observed in young and old cohorts. The scale bar is 10 μm. (C) The mean GFC diameter in each nucleolus was analyzed by taking the mean of two perpendicular measurements, and these measurements are plotted for the two age cohorts. A *t*‐test was performed, and the asterisks denotes *P* = 0.0317. Four experimental replicates were performed, and a total of > 15 follicles per group were analyzed. (D). The patterns of fibrillarin expression in oocyte nucleoli were examined in follicles isolated from reproductively young and old mice. Representative images of fibrillarin staining in the nucleoli of oocytes from young (top panel) and old (bottom) cohorts are shown from one experiment. Four experimental replicates were performed. White asterisks indicate the nucleoli that were excluded from quantification to avoid duplication. The scale bar is 10 μm. (E) Fibrillarin intensities were quantified using image j, and only a defined central region of each nucleolus was used for quantification. Mean pixel intensity from four independent replicates was normalized to the mean of the control, and the combined fold expression was determined. A *t*‐test was performed, and the asterisks denotes *P* < 0.0001.

Using these nucleolar markers, we performed a comparative analysis of nucleolar architecture in growing oocytes from reproductively young and old mice (Fig. 5 and Fig. S3). The majority of oocytes from reproductively old mice had only one nucleolus, whereas the majority from reproductively young mice had more than one (43% vs. 25%, respectively, *P* = 0.0109; Fig. S6A). No age‐associated changes in nucleolar diameter or in nucleolin distribution were documented (data not shown and Fig. S6B). Interestingly, however, we did observe prominent differences in UBTF and fibrillarin expression profiles in oocyte nucleoli between the two age cohorts (Fig. 5). A higher percentage of oocyte nucleoli from reproductively old mice contained GFCs compared to those from young mice (50% vs. 32%, respectively, *P* = 0.0143; Fig. 5A). Moreover, the GFCs in oocyte nucleoli from reproductively old mice were on average larger than those from young mice (1.883 ± 0.1294 μm vs. 1.385 ± 0.1688 μm, respectively; *P* = 0.0317; Fig. 5B,C). Fibrillarin expression was increased in nucleoli of oocytes isolated from reproductively old mice relative to young mice as evidenced by increased fluorescence intensity across all experimental replicates (Fig. 5D and Fig. S6C–F). This increase corresponded to a significant overall 1.856‐fold reproductive age‐associated increase in fibrillarin expression in oocyte nucleoli (*P* < 0.0001; Fig. 5E). Increased fibrillarin expression has been implicated in aberrant rRNA methylation and thereby the increased production of ribosomes with compromised translational fidelity (Marcel *et al*., 2013, 2015). Taken together, these observations suggest that oocyte aging is associated with architectural changes in the nucleolus that are indicative of dysregulation, including potentially increased rDNA transcription (more and larger GFCs) and aberrant methylation (increased fibrillarin expression).

We also evaluated oocyte nucleolar ultrastructure by transmission electron microscopy (TEM) on intact follicles from reproductively young and old mice (Fig. S7A and Supporting Information). The mammalian nucleolus has a tripartite organization visible by TEM composed of fibrillar centers (FC), dense fibrillar centers (DFC), and granular centers (GC) (Melese & Xue, 1995). FC and DFC are essential structural components of the nucleolus, and together, they form FC/DFC units, which are the sites of rDNA transcription and early protein processing (Melese & Xue, 1995). By TEM, FC and DFC correspond to electron‐lucent and electron‐dense regions, respectively (Fig. S7B,C; Chouinard, 1971). UBTF localizes to the FC and fibrillarin to the DFC (Song *et al*., 2009). Oocyte nucleoli from reproductively old mice had prominent FC and more densely arranged DFC, providing additional evidence of altered nucleolar structure (Fig. S7B,C).

### Advanced reproductive age is associated with increased ribosome numbers in growing oocytes

**Figure 6:**
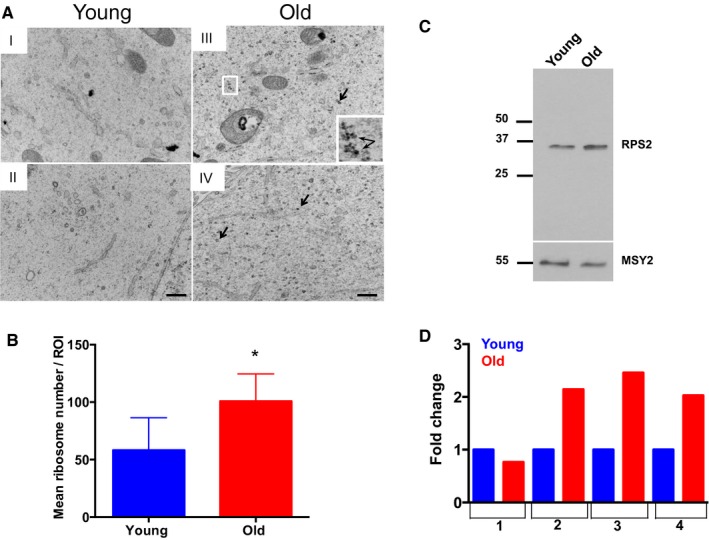
Oocytes from reproductively old mice have increased ribosome numbers. (A) Representative transmission electron microscopy (TEM) images of the cytoplasm of oocytes from reproductively young (I, II) and old (III, IV) mice are shown. Arrows and the boxed region and inset highlight ribosomes. The scale bar is 500 nm. (B) Ribosome number was quantified using image j, and the graph shows the average number of ribosomes per defined region of interest in oocytes from reproductively young and old mice. A *t*‐test was performed, and the asterisks denotes *P* = 0.0139. (C) Oocyte protein extracts from reproductively young and old cohorts were processed for immunoblot analysis with an RPS2 antibody (10 oocytes/lane). The immunoblot was reprobed with an antibody against the oocyte‐specific protein MSY‐2 as a loading control. (D) The graph shows the fold change in RPS2 expression in young and old cohorts from four independent experiments. The overall fold increase in RPS2 expression across experiments was 1.851 (*P* = 0.0627).

When performing TEM to assess the nucleolus, we were struck by dramatic and unexpected differences in the cytoplasm between oocytes from reproductively young and old mice (Fig. 6A,B). Specifically, oocytes from reproductively old mice contained noticeably more cytoplasmic ribosomes relative to young counterparts (Fig. 6A,B). Using a density threshold analysis, we determined that oocytes from reproductively young mice had 58.24 ± 10.65 ribosomes per cytoplasmic region of interest (ROI) compared to 100.9 ± 9.673 in oocytes from old mice, corresponding to a 1.732‐fold increase (*P* = 0.0139; Fig. 6B). To further validate this ribosomal number increase, we compared the expression levels of RPS2, a 40S subunit component, between oocytes from reproductively young and old mice by immunoblot analysis as levels of ribosomal proteins have been used previously as indirect measures of ribosomal number (Fig. 6C,D; Azpurua *et al*., 2013). We used oocytes isolated from early antral follicles, so that we could assess protein levels without confounding expression from somatic cells. RPS2 levels were higher in oocytes isolated from reproductively old mice compared to young mice in three of the four experimental replicates (Fig. 6D). This corresponded to an overall age‐associated increase in RPS2 expression of 1.85‐fold, which trended toward significance (Fig. 6D, *P* = 0.0627). These findings are consistent with a previous microarray study, which demonstrated increased expression of *Rps2* in fully grown oocytes isolated from reproductively old mice relative to young and further supports the observed age‐associated increase in ribosome number (Pan *et al*., 2008).

## Discussion

In this study, we used multimodal approaches to demonstrate that reproductive aging is associated with protein metabolism dysregulation during the active growth phase of the mammalian oocyte. Although follicles isolated from reproductively young and old mice appeared similar by standard light microscopy, their molecular and subcellular signatures were distinct. Oocytes from mice of advanced reproductive age had a gene expression pattern consistent with disruptions in proteostasis, and prominent age‐associated alterations in nucleolar structure and function were observed. Significant nucleolar changes included increased localization of UBTF to large GFCs and increased levels of fibrillarin expression. These changes were mirrored at the ultrastructural level, where nucleoli from old oocytes had prominent FCs and DFCs where UBTF and fibrillarin are known to be expressed, respectively (Song *et al*., 2009). These changes are significant because the architecture of the nucleolus is exquisitely tuned to its function, and the specific alterations we documented in nucleolar architecture are indicative of dysregulated function (Olson & Dundr, 2005). In fact, we observed corresponding increases in oocyte‐to‐follicle diameter ratio, ribosome protein expression, and ribosome number with age. Together, this evidence points toward nucleolar dysfunction and disrupted proteostasis as new mechanisms underlying reproductive aging.

Whether the observed changes in nucleolar architecture and ribosome production are reflected in higher levels of protein synthesis is not yet known. It is also possible that these changes affect the quality of the proteins that are produced with age. For example, similar to aged oocytes, fibrillarin is overexpressed in breast and prostate cancers in both mouse and humans (Marcel *et al*., 2013; Su *et al*., 2014). In these cancer cells, high levels of fibrillarin are associated with increased RNA Pol I activity, resulting in enhancement of ribosome biogenesis (Marcel *et al*., 2013). Moreover, dysregulated fibrillarin expression results in altered rRNA methylation, which can impact the translation accuracy and efficiency of the resulting ribosomes (Marcel *et al*., 2013). Thus, the emerging model in cancer cells is that fibrillarin overexpression is associated with an overabundance of ribosomes that produce poor quality proteins (Marcel *et al*., 2015). Ribosomes that lack translational fidelity will produce poor‐quality proteins that might fail to support developmental competence.

Across multiple mammalian tissues and organs, alterations in protein synthesis, translational fidelity, post‐translational modifications, and protein turnover can lead to the accumulation of abnormal protein products and contribute to various pathologies (Rattan, 2010; Kaushik & Cuervo, 2015). Aging is associated with loss of protein homeostasis – or proteostasis – due to deteriorating protein quality control pathways (Kaushik & Cuervo, 2015). The oocyte is likely particularly sensitive to perturbations in protein metabolism because it is one of the most translationally active cells in the body, and ribosome biogenesis and accurate protein synthesis during oogenesis are key determinants of meiotic and cytoplasmic competence (Eichenlaub‐Ritter & Peschke, 2002). Interestingly, our RNA‐Seq data suggest that follicles from reproductively young mice have more robust protein quality control mechanisms compared to old counterparts because there was significant enrichment in young follicles of GO and KEGG pathway terms for genes involved in protein synthesis, homeostasis, and modification. Thus, reduced proteostasis may underlie reproductive aging in the mammalian oocyte.

In summary, our work sheds new insight into the age‐associated decline in oocyte quality. By performing RNA‐Seq on individual follicles, we were able to make the fundamental observation that reproductive aging is a biological continuum and does not impact all mice or follicles uniformly. Not all follicles within an individual animal had the same gene expression profile, and some follicles from mice of advanced reproductive age were more similar to those from young mice. Such heterogeneity is also observed in humans where not all women of advanced reproductive age will experience difficulty conceiving, and not all eggs from an individual female will have the same developmental potential. These results, therefore, further support the use of the mouse as a model to study reproductive aging. Despite this inherent biological variation, we identified protein metabolism dysregulation as a hallmark of reproductive aging in the mammalian oocyte, which originates during the active growth phase of folliculogenesis. These findings lay a strong foundation and define an important model system for further investigating aging ribosomes and the aging translatome. Protein metabolism dysregulation represents a novel paradigm for the reproductive age‐associated decline in oocyte quality along with existing models of meiotic defects, mitochondrial dysfunction, and microenvironment alterations (Hunt & Hassold, 2008; Ben‐Meir *et al*., 2015; Briley *et al*., 2016).

## Experimental procedures

### Animals

Two age cohorts of adult CB6F1 female mice were used: 6‐ to 12‐week‐old mice (reproductively young; Envigo, Indianapolis, IN, USA) and 14‐ to 17‐month‐old mice (reproductively old; National Institute on Aging Aged Rodent Colony, Bethesda, MD, USA). 14‐ to 17‐month‐old mice were selected as the reproductively old cohort because it has been shown that a significant deterioration in egg quality and the ovarian microenvironment occurs at this age (Chiang *et al*., 2010; Briley *et al*., 2016). We acknowledge that we do not know whether the founder substrains used to produce the F1 animals in our study were identical between our young and old mouse cohorts. Although we cannot rule out confounding effects due to potential substrain variability, we think this contribution is likely to be minimal given that most reproductive aging phenotypes are conserved across strains (i.e., follicle loss, increased egg aneuploidy, increased ovarian stromal fibrosis; Chiang *et al*., 2010; Lister *et al*., 2010; Hirshfeld‐Cytron *et al*., 2011; Merriman *et al*., 2012; Briley *et al*., 2016). Prepubertal (12‐ to 13‐day‐old) CD‐1 female mice (Envigo) were used to optimize nucleolar antibody staining conditions. All mice were housed in a controlled barrier facility at the University of Kansas Medical Center's (KUMC) Research Support Facility under constant temperature, humidity, and light (12‐h light/12‐h dark). Food and water were provided *ad libitum*. All experimental protocols were approved by the Institutional Animal Care and Use Committee of the University of Kansas Medical Center and were in accordance with National Institutes of Health Guidelines.

### Tissue processing and follicle counting analysis

Ovaries were harvested from both young (*n* = 3) and old mice (*n* = 3) and were fixed in modified Davidson's fixative (Electron Microscopy Sciences, Hatfield, PA, USA) for 6 h at room temperature and then overnight at 4 °C. After fixation, these ovaries were dehydrated in a graded ethanol series and embedded in paraffin. Embedded ovaries were serially sectioned (5 μm thickness) and stained with hematoxylin and eosin (H&E) according to the standard protocols. To view entire ovary sections, individual 40× brightfield images were taken and stitched together using an EVOS FL Auto Cell Imaging system (Thermo Fisher, Waltham, MA, USA).

Follicles were counted in every fifth section throughout a single ovary for each animal based on previously published protocols (Bristol‐Gould *et al*., 2006). Follicle stages were classified according to morphological criteria, and only healthy follicles were counted (Fig. 1A and Fig. S1A). In brief, primordial follicles were classified as an oocyte that was partially surrounded by only squamous granulosa cells, and primary follicles were characterized as oocytes surrounded by a single layer of granulosa cells in which the squamous‐to‐cuboidal transition was apparent. All primordial and primary follicles were counted irrespective of whether the oocyte nucleus was visible in the section. Secondary follicles contained a larger oocyte surrounded by more than one layer of granulosa cells, and transitioning secondary follicles contained small spaces indicative of early cellular rearrangements for antrum formation. Early antral follicles contained a visible antrum, and antral follicles were the largest follicles with prominent antral spaces. For larger follicles (secondary through antral stages), only those with an oocyte nucleus present in the section were counted to avoid double counting. The total follicle count for each stage per ovary was then divided by the total number of sections counted per ovary. The average number of follicles per section for each follicle stage was reported. To examine follicle growth dynamics, mean follicle and oocyte diameter measurements were performed for each follicle. The average of two perpendicular measurements was taken from basement membrane to basement membrane (follicle) or from plasma membrane to plasma membrane (oocyte; Fig. 1C).

### Early growing follicle and oocyte isolation

Ovaries were harvested from reproductively young and old mice in Leibovitz's L‐15 medium (Life Technologies Corporation, Grand Island, NY, USA) supplemented with 3 mg/mL polyvinylpyrrolidone (Sigma‐Aldrich, St. Louis, MO, USA) and 0.5% Pen Strep (Life Technologies; L15/PVP). Ovaries were disrupted mechanically using insulin syringes and late primary to transitioning secondary‐stage follicles were identified based on size and morphology and collected. For short‐term culture (maximum of 40 min), follicles were cultured in CZB media (EMD Millipore, Billerica, MA, USA) overlaid with mineral oil in a humidified atmosphere of 5% CO_2_ in air at 37 °C. Follicles were imaged using the EVOS FL Auto Cell Imaging system, and the mean diameter was determined based on two perpendicular measurements from basement membrane to basement membrane. These follicles were used for downstream experiments as described below. For RPS2 immunoblot analysis, cumulus–oocyte complexes (COCs) from small antral follicles were released following mechanical disruption of the tissue using insulin syringes. COCs were collected in L15/PVP supplemented with 2.5 μm milrinone (Sigma‐Aldrich) to maintain meiotic arrest. Oocytes within the COCs were mechanically stripped free of cumulus cells, and denuded oocytes were immediately snap‐frozen.

### RNA sequencing and analysis

Follicles were isolated from reproductively young (*n* = 5) and old CB6F1 (*n* = 4) mice as mentioned above. Isolated follicles were placed individually in 5 μL droplets of L15/PVP under oil and imaged immediately (Fig. S6A,B). Individual follicles were snap‐frozen in 3.5 μL 2× reaction buffer supplied in the cDNA synthesis reagents. For each animal, the total time that elapsed between mouse euthanasia and snap‐frozen samples did not exceed 30 min. Construction of cDNA was performed using the Smart‐Seq v4 Ultra Low Input RNA kit (Clontech Laboratories Inc, Mountain View, CA, USA) with 14 cycles of PCR according to the manufacturer's instructions. Quality and yield of cDNA was assessed using an Agilent 2100 Bioanalyzer (Agilent Technologies, Santa Clara, CA, USA). Adapter‐ligated libraries were constructed from 1 ng cDNA using the Nextera XT kit (Illumina Inc, San Diego, CA, USA) according to the manufacturer's instructions. Resulting libraries were checked for quality and quantity using the Agilent 2100 Bioanalyzer and Qubit Fluorometer (Thermo Fisher Scientific). Libraries were pooled, requantified, and sequenced as 50‐bp single read on the Illumina HiSeq 2500 instrument using the hiseq control Software 2.2.58, Illumina, Inc, San Diego, CA, USA. Following sequencing, Illumina Primary Analysis version rta 1.18.64, Illumina, Inc, San Diego, CA, USA and Secondary Analysis version casava‐1.8.2, Illumina, Inc, San Diego, CA, USA were run to demultiplex reads for all libraries and generate FASTQ files. Data were obtained for 24 follicles in total, with 10 derived from reproductively old mice and 14 from young mice. For each library, a minimum of 20 million reads were collected. Read sets averaged 43% unique sequence with 90% alignability. Reads were aligned to UCSC mm10 genome (http://genome.ucsc.edu/) with star 2.4.2a (Dobin *et al*., 2013), using gene models from Ensembl 80 (http://www.ensembl.org). Read counts calculated by star were analyzed in r with the edger package (Robinson *et al*., 2010), default methods, contrasting young vs. old, treating all samples as replicates. As mentioned above, only genes with Ensembl biotype ‘protein coding’, ‘pseudogene’, or ‘lncRNA’ were analyzed, and had to have at least four counts in any sample. Of 23 409 such genes, at FDR ≤ 0.05, 411 of 1553 were significantly up‐/downregulated.

Expression value of young samples was compared to public oocyte data [(Veselovska *et al*., 2015), GEO:GSE70116] to help determine which genes were more likely to be expressed in the oocyte vs. the granulosa cells. The Growing‐Oocyte 2 sample (GO2) was the most similar in stage to our young samples. Based on the log2‐RPKM distribution, we chose 1.75 as the expression threshold for GO2. We found that the young‐sample mean log2 RPKM value of 5.25 could serve meaning a simple classifier for oocytic expression, having a true‐positive rate of 78%, as 78% of genes above this value are expressed in GO2 and 78% of genes below this value are unexpressed in GO2. Genes considered as likely to have been expressed in oocyte or granulosa cells were classified in this way. Raw data have been submitted to ArrayExpress under accession E‐MTAB‐5952.

### Immunofluorescence, confocal microscopy, and image analysis

To optimize nucleolar antibody staining conditions, oocytes from prepubertal mice were collected as previously described and were fixed either in 2% paraformaldehyde (PFA; Electron Microscopy Sciences) or in 2% PFA containing 0.1% Triton X‐100 for 40–50 min (Stein, 2009; Fig. S4). Oocytes were incubated in blocking buffer (1× phosphate‐buffered saline; PBS supplemented with 3 mg/mL bovine serum albumin; BSA, 0.01% Tween‐20, and 0.02% sodium azide) overnight at 4 °C and permeabilized for 15 min in permeabilization buffer (1× PBS supplemented with 3 mg/mL BSA, 0.1% Triton X‐100, and 0.02% sodium azide). A subset of oocytes that had been fixed in 2% PFA were permeabilized for 1 h instead of 15 min. Following permeabilization, all oocytes were rinsed twice in blocking buffer. Oocytes were incubated in primary antibodies overnight at 4 °C. Primary antibodies included mouse anti‐UBTF (Cat # H00007343‐M01; Abnova, Taipei City, Taiwan), rabbit anti‐fibrillarin (Cat # 2639; Cell Signaling, Danvers, MA, USA), and rabbit anti‐nucleolin (Cat # ab70493; Abcam, Cambridge, MA, USA). A dilution series for each antibody was tested, and they were ultimately used at final concentrations/dilutions of 4 μg/mL, 1:50, and 0.8 μg/mL, respectively. Oocytes were rinsed three times in blocking buffer and then incubated in secondary antibody for 1 h at room temperature using a 1:100 dilution of goat anti‐mouse Alexa Fluor 488 or goat anti‐rabbit Alexa Fluor 488 (Life Technologies). Oocytes were then washed three times in blocking buffer and mounted in Vectashield containing DAPI (4′, 6‐diamidino‐2‐phenylindole; Vector Labs, Burlingame, CA, USA). The fixation and staining conditions optimized in the oocyte were then applied to the intact follicle. Follicles were isolated as described above from the ovaries of reproductively young (*n* = 5) and old mice (*n* = 11) and fixed in 2% PFA for 40 min at room temperature. Follicles from young and old mice were isolated and processed in parallel.

Samples were imaged using a TCS SPE laser scanning confocal microscope (Leica Microsystems, Buffalo Grove, IL, USA) equipped with 40× oil immersion objective and Argon (488 nm) and near‐UV (405 nm) laser lines. Images were acquired as Z‐stacks (1 μm step size). Images were analyzed using LAS AF (Leica Microsystems) and image j (National Institutes of Health, Bethesda, MD, USA) software. For fibrillarin analysis, the laser power and gain values were kept constant throughout the scanning, so intensities between young and old samples could be compared. To quantify fibrillarin intensity, mean pixel values in a constant defined region of interest (ROI) in the nucleolus of each oocyte were determined using image j software. The background intensities were subtracted and the corrected mean intensities were normalized to young controls. The fold change value was determined by dividing the normalized intensities of old cohorts over normalized intensities of young cohorts.

### Transmission Electron Microscopy (TEM) and ribosome number quantification

All materials and reagents for TEM were obtained from Electron Microscopy Sciences. Follicles were isolated as described above (*n* = 2 young and *n* = 2 old mice) and were fixed in 2% glutaraldehyde in 0.1 m sodium cacodylate buffer. Follicles were spun down in microfuge tubes for this step as well as between each step that involved a solution change. All of the steps were carried out in the microfuge tubes. Follicles were rinsed in fresh cacodylate buffer for 5 min. They were then postfixed in 1% buffered osmium tetroxide for 1 h. Follicles were rinsed in three exchanges of deionized distilled water. The samples were then dehydrated through a graded series of ethanol as follows: 50%, 70%, 80%, 95%, and 100% twice, for 10 min during each step. After dehydration, the samples were incubated in propylene oxide for 20 min, then into a mixture of half volume propylene oxide and half volume Embed 812 medium overnight. The half‐and‐half mixture was removed the next day and 100% Embed 812 medium was added to the tube and samples were incubated for 1 h. Resin was removed and replaced with fresh Embed 812 medium and placed in a 65 °C oven to polymerize overnight. Samples were sectioned using a Diatome diamond knife on a Leica UC‐7 ultra microtome (Leica Microsystems). Sections were cut at 1 μm thickness until the nucleus of the oocyte was located, and then, sections were cut at 70 nm and picked up on 300‐mesh thin bar grids. Section contrast was achieved with 3% uranyl acetate and Sato's lead stain. Samples were viewed with a JEM 1400 TEM (J.E.O.L. USA, Peabody, MA, USA) at 100 kv. For ribosome analysis, we identified these organelles by their size (20–30 nm in diameter) and morphology. The number of ribosomes was quantified in defined region of interests (ROIs) in the oocyte cytoplasm using image j (http://imagej.net/Particle_Analysis). Regions that were relatively devoid of other organelles were selected for analysis, and to ensure that structurally equivalent areas of the oocyte were examined, we analyzed three independent ROIs per oocyte that spanned the region between the nucleus and cortex. The number of ribosomes in these three distinct ROIs was averaged together per oocyte.

### Immunoblot analysis

Protein extracts of equal numbers of oocytes (10 oocytes per lane) isolated from COCs from young and old mice were separated by gel electrophoresis using 4–15% Mini‐Protean TGX gels (Bio‐Rad, Hercules, CA, USA) under standard reducing conditions. Proteins were transferred to PVDF blotting membrane (GE Health Care Life Science, Pittsburg, PA, USA) at 100 volts for 1 h. Membranes were blocked at room temperature with gentle rocking for 4 h in 3% ECL Prime Blocking Agent (GE Health Care Life Sciences) diluted in Tris‐buffered saline containing 0.1% V/V Tween‐20 (TBST). Blots were then incubated in rabbit anti‐RPS2 antibody (Cat # ab155961; Abcam) at a 1:1000 dilution overnight at 4 °C. Blots were washed three times in TBST and incubated for 1 h at room temperature in a 1:10 000 dilution of horseradish peroxidase‐labeled donkey anti‐rabbit secondary antibody (GE Health Care Life Sciences). Blots were washed three times in TBST followed by chemiluminescence detection using the ECL prime detection kit according to the manufacturer's instructions (GE Health Care Life Sciences). Following RPS2 detection, the blots were washed briefly in TBST and reprobed with a 1:5000 dilution of an MSY2 antibody (generous gift of R. Schultz, University of Pennsylvania), overnight at 4 °C. Secondary antibody incubation and chemiluminescence detection were performed as described above. Densitometry analysis was performed using image j software to evaluate the differences in RPS2 expression in oocytes from reproductively young and old mice. Mean pixel intensities for RPS2 and MSY2 bands were determined following mean background subtraction. Because equal numbers of oocytes were loaded per lane, RPS2 mean pixel intensity values were normalized to MSY2 mean pixel intensity values, as MSY2 is a highly abundant oocyte‐specific protein in the ovary (Yu *et al*., 2001). The fold change value was determined by dividing the normalized RPS2 intensities of old cohorts over normalized RPS2 intensities of young cohorts.

### Statistical analysis

Significant changes between groups were analyzed by either Students’ *t*‐test or chi‐square analysis. *P* values < 0.05 were considered statistically significant. An association between two parameters was analyzed by performing either Spearman's or Pearson's correlation. Statistical analysis was performed using graphpad prism Software version 6.0f: GraphPad Software, Inc. (La Jolla, CA, USA).

## Conflict of interest

The authors have no conflict of interest to report.

## Funding

This work was supported by the Centers of Biomedical Research Excellence (P20 GM104936, F.E.D). In addition, summer student research for this project was supported by the Kansas Institutional Development Award (IDeA) (P20 GM103418, J.M.K). The Electron Microscopy Research Laboratory and Anatomy/COBRE Confocal Imaging Facility at KUMC are supported in part by NIH COBRE P20GM104936, and the JEOL JEM‐1400 TEM used in the study was purchased with funds from S10RR027564. The Histology Core at KUMC is supported by P30 HD002528 (Kansas IDDRC).

## Author contributions

S.J., J.L.G, and F.E.D. designed the experiments and wrote the manuscript. S.J., A.P., J.M.K, B.F, and F.E.D. performed experiments. All authors participated in data analysis and read and approved the manuscript.

## Supporting information


**Fig. S1** Coordinated oocyte and follicle growth is altered with advanced reproductive age.
**Fig. S2** Additional RNA‐Seq data analysis on follicles from reproductively young and old mice.
**Fig. S3** Comparative analysis of oocyte nucleolar markers was performed in similar populations of intact early growing follicles.
**Fig. S4** Cross‐linking with 2% PFA results in optimal nucleolar protein localization.
**Fig. S5** Nucleolar proteins have distinct localization patterns in the growing oocyte.
**Fig. S6** Additional comparative analysis of nucleolus parameters in oocytes from reproductively young and old mice.
**Fig. S7.** Reproductive age‐associated differences exist in the oocyte nucleolus at the ultra‐structural level.

**File S1** EdgeR data for significant differentially expressed genes (MS_sig.edgeR. genes.xlsx).

**File S2** Functional data for hypervariable genes (MS_hypervariable.analysis.xlsx).
