# Supplementary material for: Age‐associated dysregulation of protein metabolism in the mammalian oocyte
Source: Aging Cell. 2017 Oct 10;16(6):1381–93. doi: 10.1111/acel.12676 (PMC5676066; doi:10.1111/acel.12676)
Supplement: Supplementary file 1 — Fig. S1 Coordinated oocyte and follicle growth is altered with advanced reproductive age. Fig. S2 Additional RNA‐Seq data analysis on follicles from reproductively young and old mice. Fig. S3 Comparative analysis of oocyte nucleolar markers was performed in similar populations of intact early growing follicles. Fig. S4 Cross‐linking with 2% PFA results in optimal nucleolar protein localization. Fig. S5 Nucleolar proteins have distinct localization patterns in the growing oocyte. Fig. S6 Additional comparative analysis of nucleolus parameters in oocytes from reproductively young and old mice. Fig. S7. Reproductive age‐associated differences exist in the oocyte nucleolus at the ultra‐structural level. [file ACEL-16-1381-s001.pptx]

## Slide 1
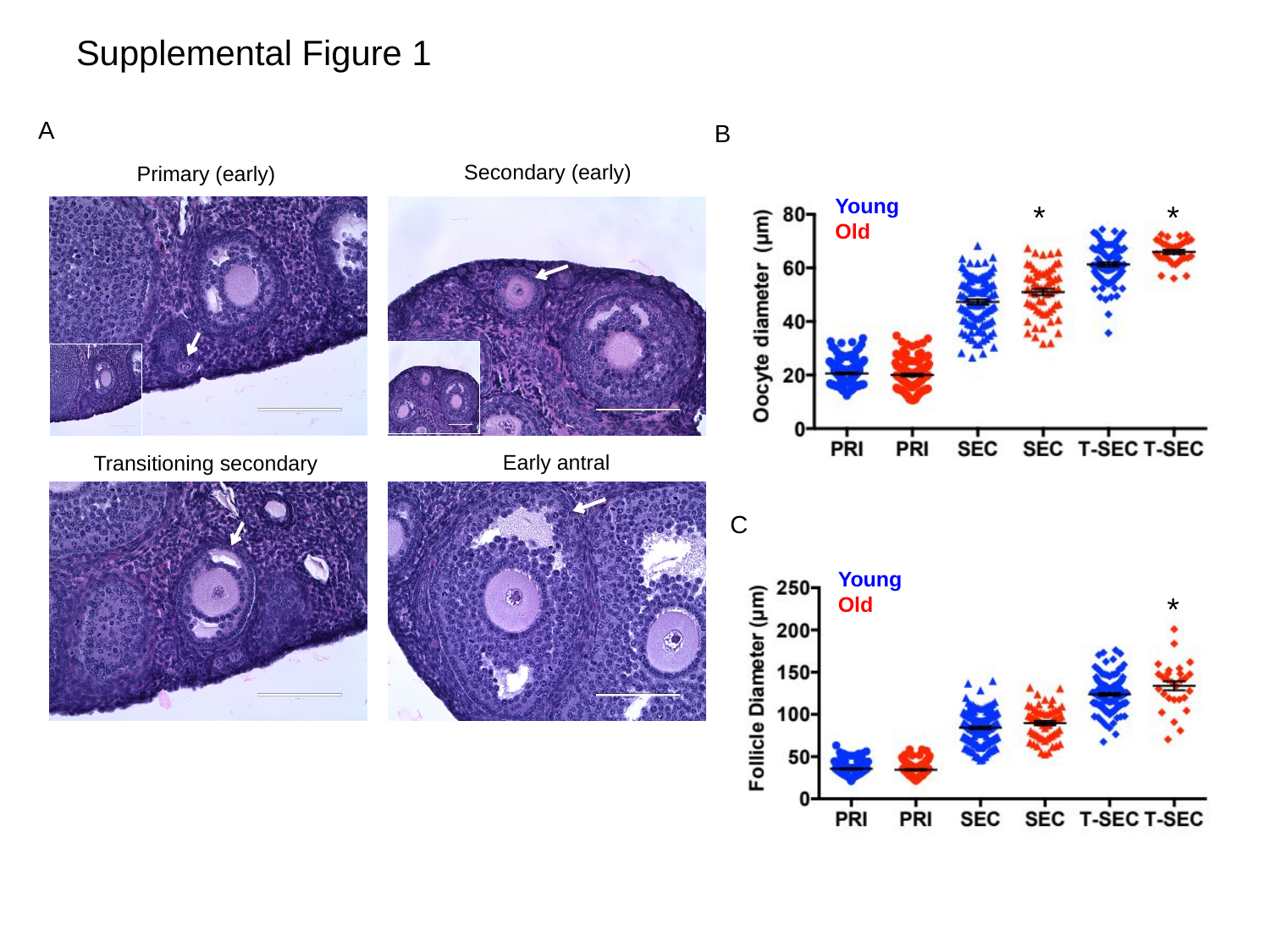

# Supplemental Figure 1
A
B
Secondary (early)
Primary (early)
Young
Old
*
*
Early antral
Transitioning secondary
C
C
Young
Old
*

## Slide 2
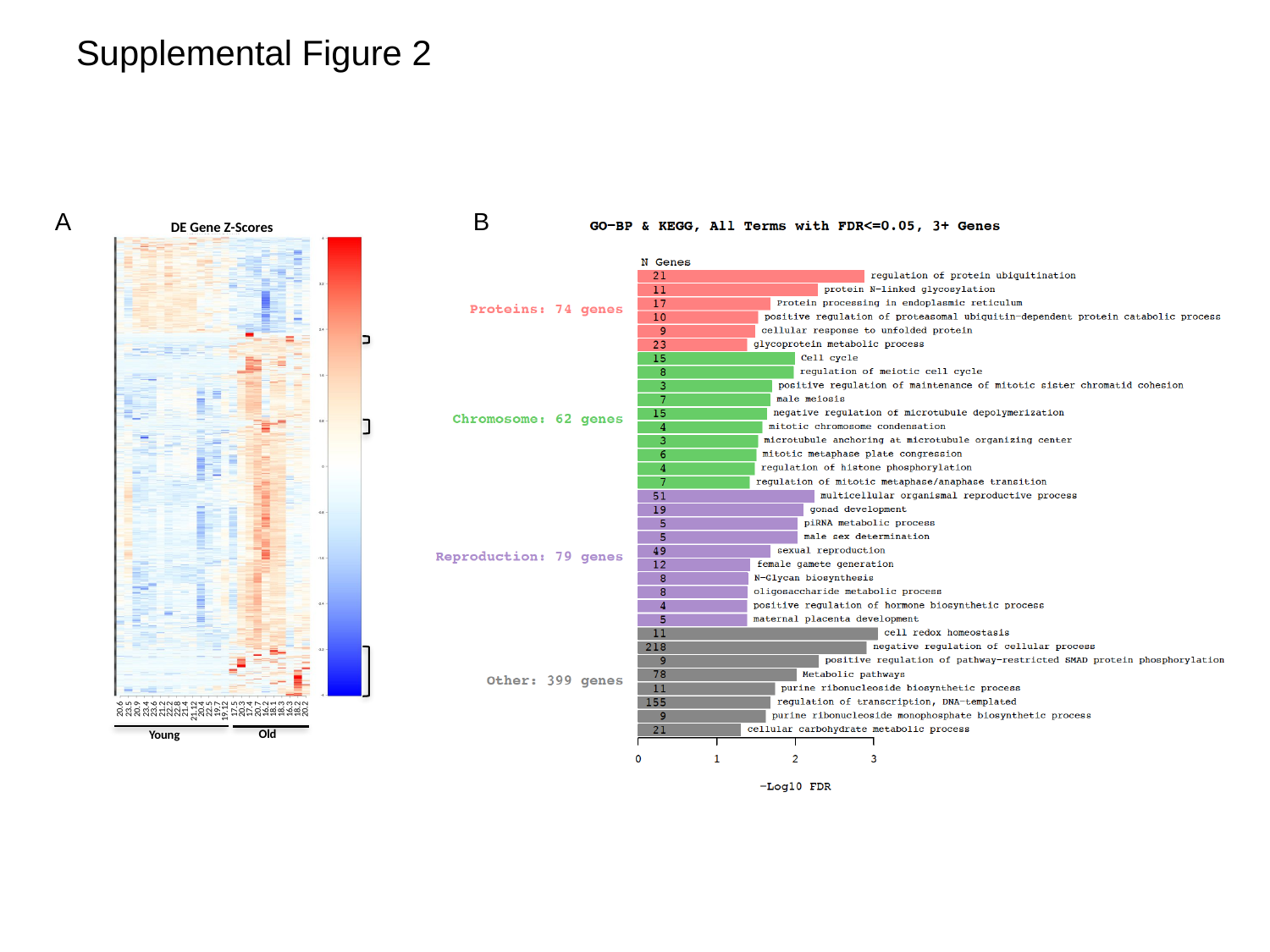

# Supplemental Figure 2
A
B
DE Gene Z-Scores
20.6
23.5
20.9
23.4
23.6
21.2
22.2
22.8
21.4
20.4
22.5
19.7
17.5
20.3
17.4
20.7
16.2
18.1
18.3
16.3
18.2
20.2
21.12
19.12
Old
Young

## Slide 3
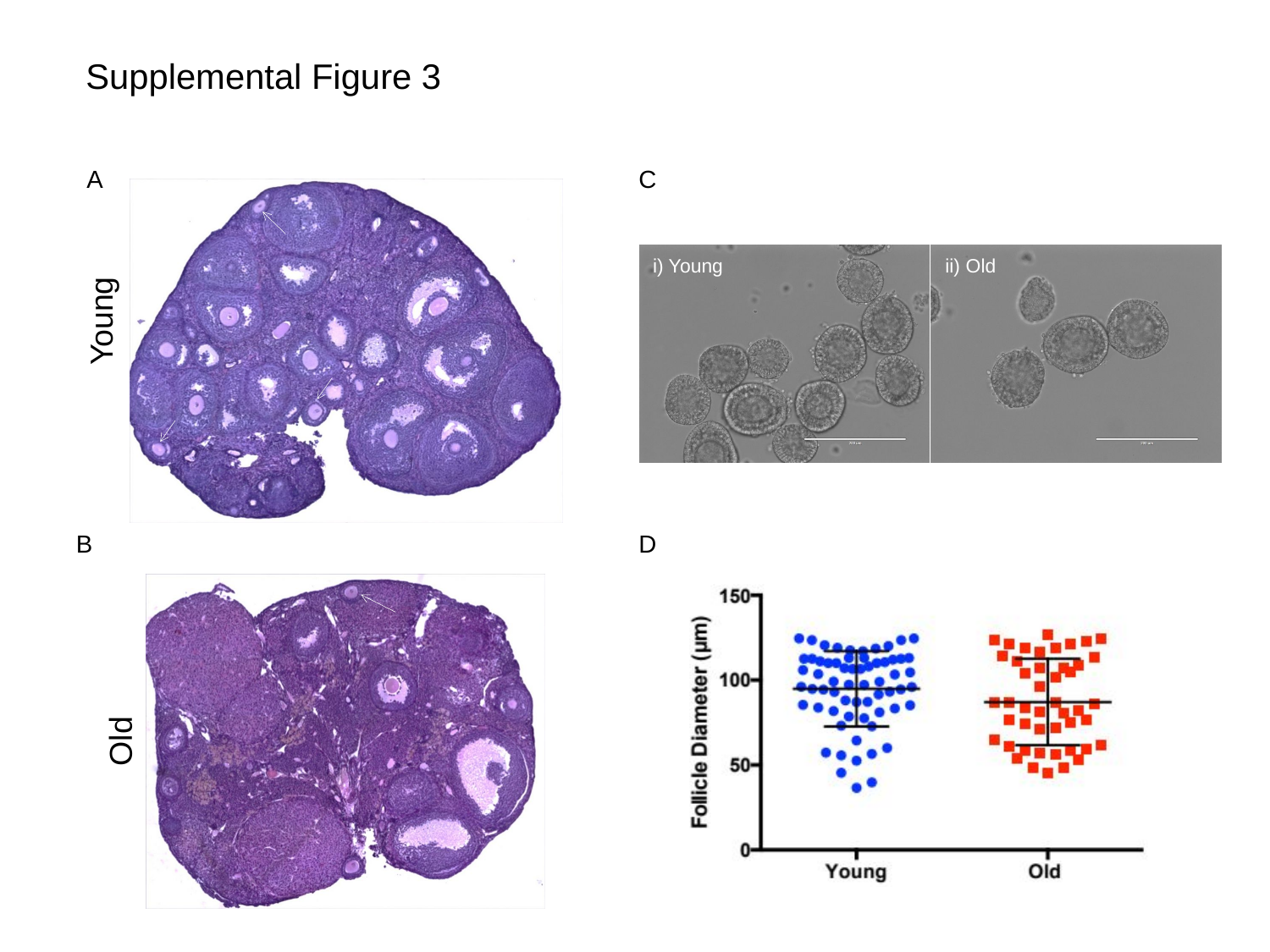

# Supplemental Figure 3
A
C
i) Young
ii) Old
Young
B
D
Old

## Slide 4
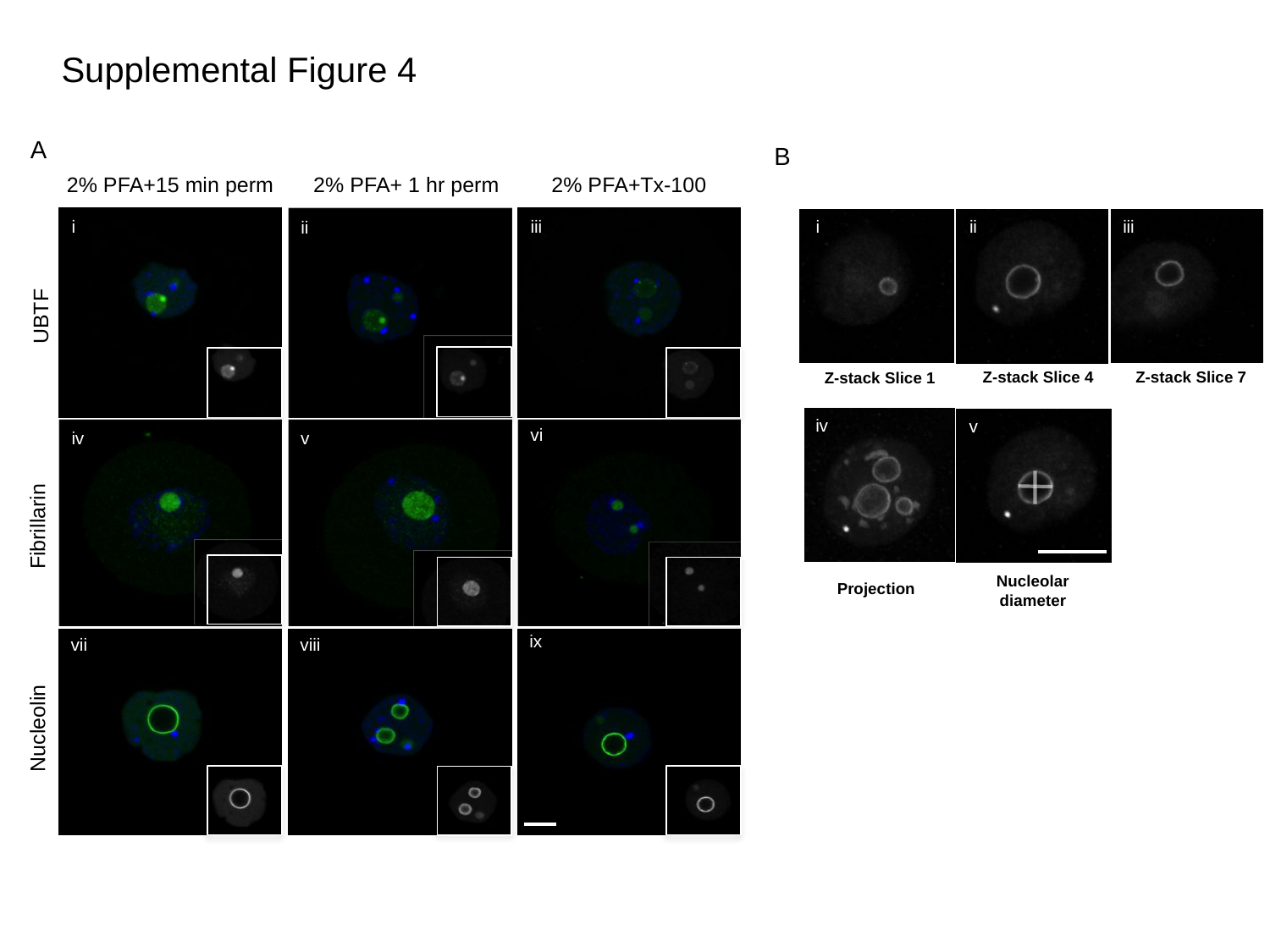

# Supplemental Figure 4
A
B
2% PFA+15 min perm
2% PFA+ 1 hr perm
2% PFA+Tx-100
Fibrillarin
Nucleolin
UBTF
i
iii
ii
vi
iv
v
ix
vii
viii
i
ii
iii
A
Z-stack Slice 4
Z-stack Slice 7
Z-stack Slice 1
iv
Projection
v
Nucleolar diameter

## Slide 5
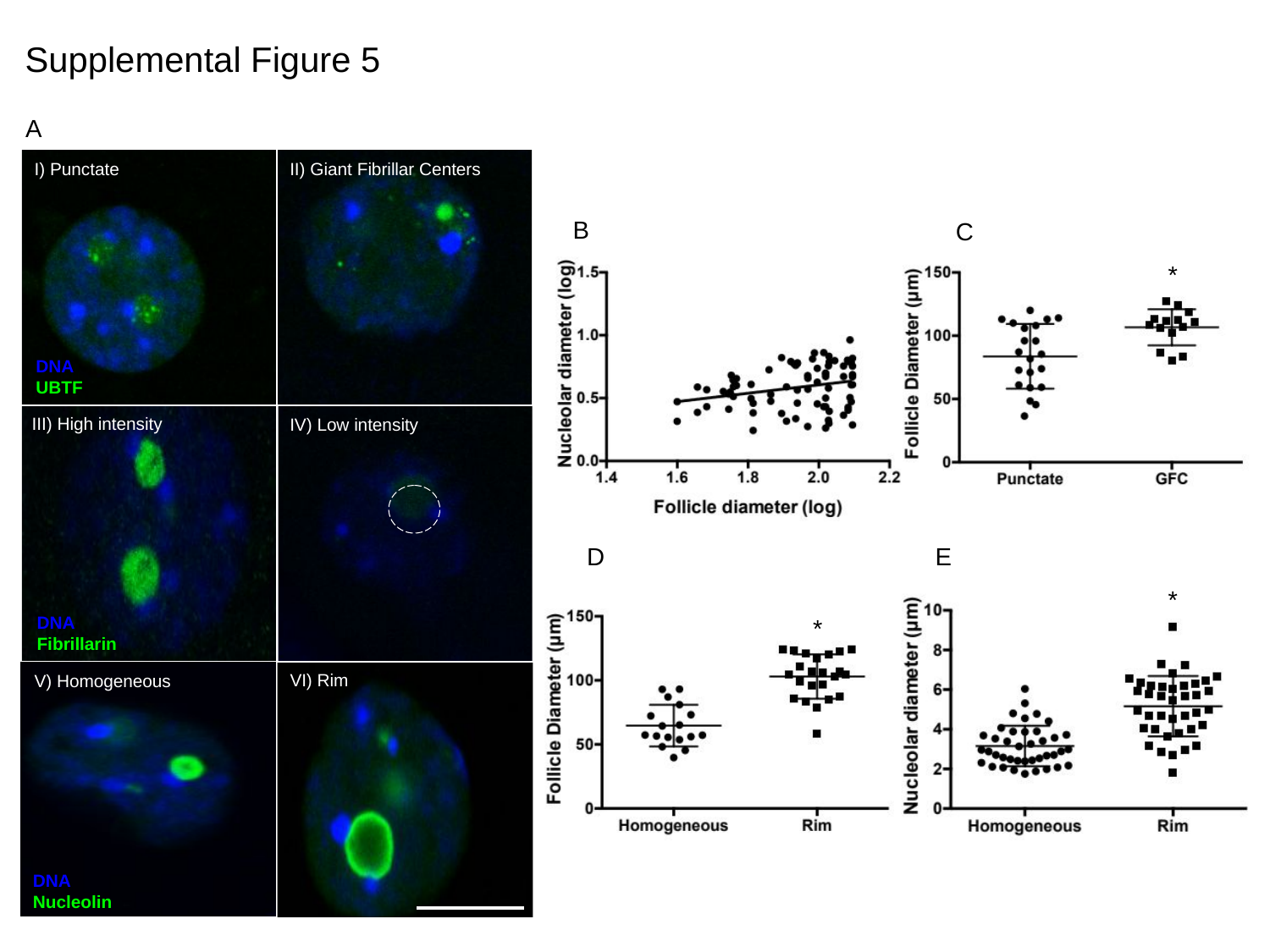

Supplemental Figure 5
A
I) Punctate
II) Giant Fibrillar Centers
DNA
UBTF
III) High intensity
IV) Low intensity
DNA
Fibrillarin
VI) Rim
V) Homogeneous
DNA
Nucleolin
B
C
*
D
E
*
*

## Slide 6
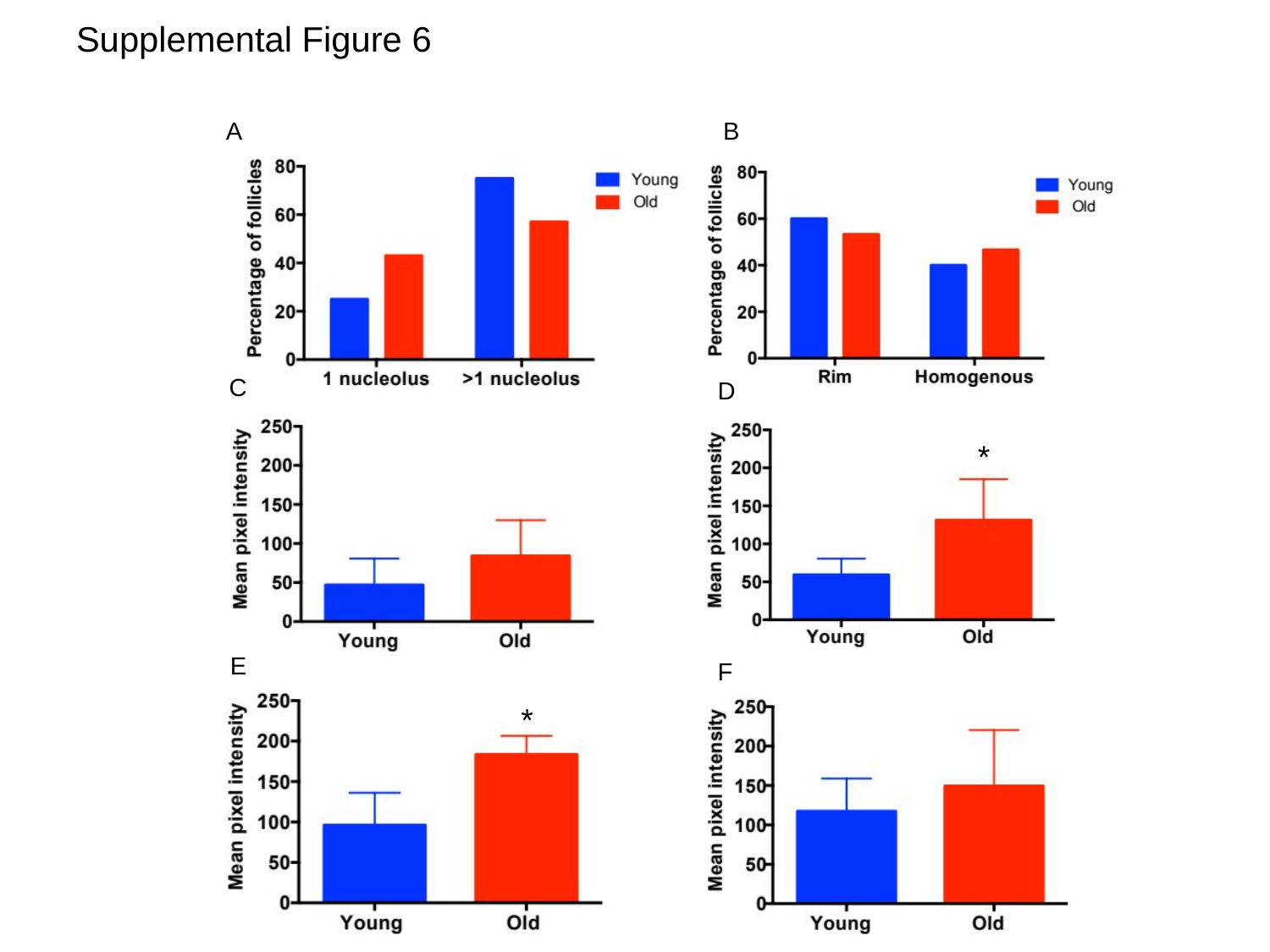

# Supplemental Figure 6
A
B
C
D
*
E
F
*

## Slide 7
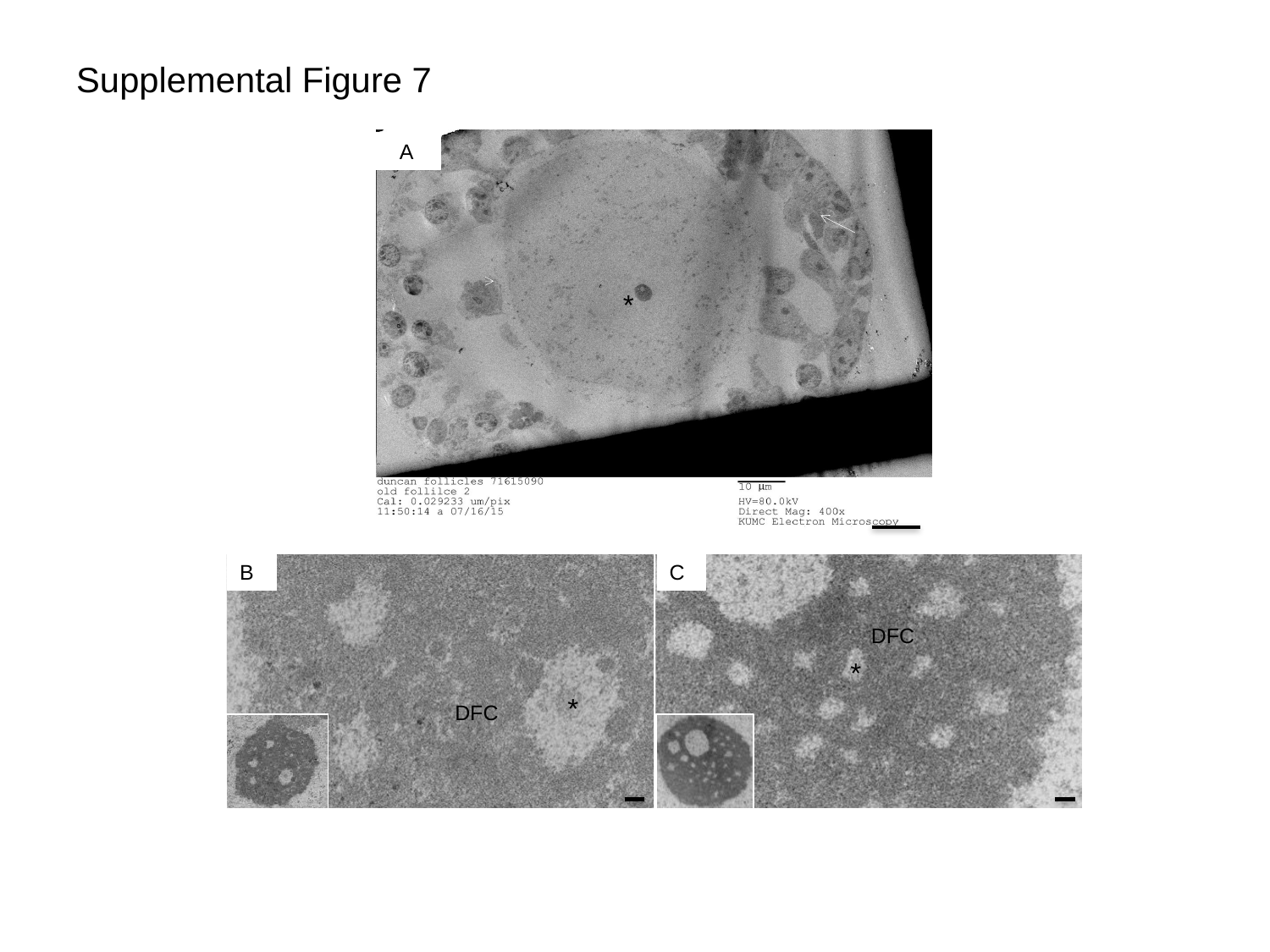

# Supplemental Figure 7
 A
*
B
C
DFC
*
*
DFC
