# Supplementary material for: Age‐associated dysregulation of protein metabolism in the mammalian oocyte
Source: Aging Cell. 2017 Oct 10;16(6):1381–93. doi: 10.1111/acel.12676 (PMC5676066; doi:10.1111/acel.12676)
Supplement: Supplementary file 2 [file ACEL-16-1381-s002.docx]

**Supporting Information:**

**File 1. EdgeR data for significant differentially expressed genes** (MS_sig.edgeR. genes.xlsx)

**File 2. Functional data for hypervariable genes** (MS_hypervariable.analysis.xlsx)

**Figure Legends**

**Supplemental Figure 1.** **Coordinated oocyte and follicle growth is altered with advanced reproductive age.** (A) H & E stained ovarian sections highlight additional follicle classifications according to histologic morphology (see Figure 1 for additional images). Arrows point to the particular follicle class in the ovarian section. Insets show magnified images of the follicles highlighted by the arrows. The scale bar is 100 μm. (B) Mean oocyte and (C) follicle diameters for each follicle class were measured from H & E stained ovarian tissue sections from reproductively young and old mice, and the diameters are plotted (PRD; primordial, PRI; primary, SEC; secondary, T-SEC; transitioning secondary). A minimum of 50 follicles from each follicle class was measured from each young (n=3) and old (n=3) mouse. Black lines denote the mean with SEM. A one-way ANOVA was performed, and the asterisks denotes P < 0.0001.

**Supplemental Figure 2. Additional RNA-Seq data analysis on follicles from reproductively young and old mice.** (A) A heat map showing age-associated differential gene expression. Each row represents the expression pattern of a single gene across multiple samples. Each column represents the expression pattern from a single follicle, as annotated at the bottom. The 14 young follicles are shown on the left side and the 10 old on the right. The relative expression levels are indicated by the look up table on the heat bar. Brackets indicate genes with hypervariable expression. (B) Biological Pathway GO terms from the differentially expressed genes assigned to the oocyte.

**Supplemental Figure 3. Comparative analysis of oocyte nucleolar markers was performed in similar populations of intact early growing follicles**. Representative H & E stained histological sections of ovaries from reproductively (A) young (6-12 weeks old) and (B) old (17-17 months old) CB6F1 mice. Arrows highlight the population of secondary follicles that were isolated and used for downstream experiments. (C) Representative isolated secondary follicles from young (i) and old (ii) cohorts were imaged by brightfield microscopy. The scale bar is 200 μm. Follicle diameters were determined by taking the mean of two perpendicular measurements, and the results are plotted in (D). A t-test shows that the follicles from young and old cohorts are similar in size (P = 0.09).

**Supplemental Figure 4. Cross-linking with 2% PFA results in optimal nucleolar protein localization**. (A) Secondary stage oocytes from pre-pubertal mice were fixed in either 2% PFA or 2% PFA with 0.1% TX-100, permeabilized for either 15 min or 1 hr, and then stained with antibodies against UBTF, fibrillarin and nucleolin. (i-ix) are merged confocal images displaying DNA (blue) and nucleolar marker staining (green). Insets show the grey scale image of the nucleoli stained for each respective marker. The scale bar is 10 μm. (B) To assess nucleolar number and diameter, follicles were stained with the nucleolin antibody, and the total number of nucleoli in each oocyte was determined by analysis of optical confocal sections. (i-iv) show an example of an oocyte containing three nucleoli. (v) The diameter of each nucleolus was determined by taking the mean of two perpendicular measurements (white lines). The scale bar is 10 μm.

**Supplemental Figure 5**. **Nucleolar proteins have distinct localization patterns in the growing oocyte**. (A) Representative confocal microscopy images of whole mount immunofluorescence performed on oocytes using specific antibodies for Upstream Binding Transcription Factor (UBTF), fibrillarin and nucleolin. Oocyte nucleoli showing UBTF expression in (I) punctate and (II) Giant Fibrillar Center (GFC) patterns. The arrow highlights a GFC. Oocyte nucleoli showing the spectrum of fibrillarin staining intensities with representative nucleoli showing (III) high and (IV) low intensity signals. The perimeter of the nucleolus with low intensity staining is highlighted with a dashed circle. Oocyte nucleoli with distinct nucleolin localization in (V) homogeneous and (VI) rim conformations. The scale bar is 10 μm. (B) Log-transformed values are plotted for nucleolar and follicle diameter. Spearman’s correlation (P = 0.0277) and linear regression were performed (R^2^ = 0.06931). (C) Oocytes stained with a UBTF-specific antibody were analyzed for GFC and punctate patterns, and the nucleolar configuration was plotted against follicle diameter. A t-test was performed and the asterisks denotes P = 0.0045. (D, E) Oocytes stained with a nucleolin-specific antibody were analyzed for homogenous and rim patterns. The nucleolar configuration was plotted against either (D) follicle or (E) nucleolar diameter as determined by taking the mean of two perpendicular measurements of the nucleolin-defined nucleolar perimeter. A t-test was performed, and the asterisks denotes P < 0.0001 in both (D) and (E).

**Supplemental Figure 6.** **Additional comparative analysis of nucleolus parameters in oocytes from reproductively young and old mice.** (A) The total number of nucleoli in oocytes from reproductively young and old mice was determined by analysis of optical confocal sections of nucleolin-stained follicles. The graph of the percentage of follicles containing an oocyte with 1 nucleolus or > 1 nucleolus shows that a significant proportion of oocytes from reproductively old mice have a single nucleolus (Chi-square analysis, P = 0.0072). (B) This graph shows the percentage of follicles in young and old cohorts that had oocytes with nucleolin in either the rim or homogeneous conformations. (C-F) Follicles were labeled with the fibrillarin antibody (see Figure 3D), and mean pixel intensities in oocyte nucleoli were quantified using Image J. Data are from 4 independent experiments. A t-test was performed, and the asterisks denotes P = 0.001 and P < 0.0001 for (D) and (E), respectively.

**Supplemental Figure 7. Reproductive age-associated differences exist in the oocyte nucleolus at the ultra-structural level.** (A) A representative transmission electron microscopy image (400X magnification) of an intact secondary follicle with a central oocyte (arrowhead) containing a prominent nucleolus (asterisks) and surrounding granulosa cells (arrow). The scale bar is 10 μm. Representative 20,000X magnification images of oocyte nucleoli from reproductively (A) young and (B) old mice are shown. Asterisks highlight fibrillar centers, which are void of electron density. Dense Fibrillar Centers (DFC), in contrast, correspond to regions of high electron density. The scale bar is 100 nm. Insets show nucleoli at 3000X magnification.
